# Integrating Case-Based Learning (CBL) and Concept Mapping (CM) enhances learning outcomes in medical biochemistry

**DOI:** 10.1186/s12909-026-08906-4

**Published:** 2026-02-26

**Authors:** Taimei Zhou, Haiying Wang, Xinglin Jiang

**Affiliations:** School of Laboratory Medicine, Hunan University of Medicine, Huaihua, Hunan 418000 China

**Keywords:** Case-based learning, Concept mapping, Meaningful learning, Medical biochemistry

## Abstract

**Background:**

Teaching biochemistry poses significant challenges in undergraduate medical education. Case-based learning (CBL) and concept mapping (CM) are two widely recognized instructional strategies in medical education. This study aimed to integrate CBL with CM in biochemistry teaching and evaluate the combined effects of these strategies on medical students' learning outcomes, compared to traditional didactic lectures.

**Methods:**

This study included 60 second-year medical students from Hunan University of Medicine (China) in their first semester. Participants were randomly assigned to two groups: a control group (*n* = 30) that received traditional didactic lectures, and an innovation group (*n* = 30) that participated in a CBL- and CM-based program. A final examination and Likert-scale questionnaires were used to evaluate the effectiveness and potential advantages of the innovative approach compared to conventional lectures.

**Results:**

Students who participated in the innovative program achieved significantly higher final examination scores than those who received traditional didactic lectures. The majority of students enjoyed using CBL combined with CM, with their overall preference for this combined approach scoring 4.93 out of 5. Notably, the highest-rated aspect (4.97/5) was the combination's effectiveness in enhancing logical thinking abilities, while its role in bridging the gap between basic science and clinical practice also received a high score of 4.83/5.

**Conclusion:**

The innovative program integrating CBL and CM not only led to higher academic performance compared to the traditional course but also received overwhelmingly positive feedback from students. They particularly valued the combined use of CBL and CM for enhancing their logical thinking skills and bridging the gap between basic science and clinical practice.

## Background

Biochemistry is a cornerstone of medical education, yet it is widely regarded as one of the most challenging subjects, with a notably high failure rate. Many students experience anxiety and frustration due to its highly abstract concepts, intricate biochemical reaction mechanisms, and complex metabolic pathways.

Concept mapping (CM), the process of constructing hierarchical visual representations of knowledge, requires learners to intentionally link and differentiate concepts [1]. Rooted in Ausubel′s assimilation theory of meaningful learning, CM has been demonstrated to effectively engage learners and promote the integration of knowledge [2–4]. To investigate the impact of CM on biochemistry learning outcomes, we integrated CM into the biochemistry curriculum for clinical medicine students during their first semester of sophomore year, and results demonstrated that CM significantly enhanced biochemistry learning following a semester-long intervention. Notably, CM proved particularly effective in facilitating knowledge integration and promoting a deeper understanding of course material [5]. Despite its advantages, the CM approach was relatively ineffective in connecting fundamental biochemistry concepts to clinical applications. As a cornerstone bridging basic science and clinical practice, biochemistry should be taught and learned through methods that actively facilitate the translation of theoretical knowledge into real-world clinical applications. Another effective tool must therefore be introduced alongside CM to address this limitation.

Case-based learning (CBL) is a learner-centered approach that guides students' learning and exploration through real-world clinical scenarios. It is well established that CBL enhances medical students' performance, clinical skills, and the integration of theory with practice [6]. Given these benefits, CBL appears to be highly complementary to CM in medical education. However, there is limited research on the combined use of CBL and CM in medical biochemistry education. To address this gap, this study aims to: 1) design and implement an integrated CBL-CM curriculum for clinical biochemistry; 2) assess the efficacy of this innovative curriculum by comparing it with traditional didactic instruction; and 3) explore medical students' perceptions regarding the usefulness of the combined CBL-CM approach in learning biochemistry.

## Subjects and methods

### Participants

Sixty second-year undergraduate students in clinical medicine, enrolled in the 2022–2023 academic year and attended the biochemistry course, were randomly assigned to two groups. All participants attended identical didactic lectures delivered by the same instructor during the first five units of biochemistry curriculum. After completing the fifth unit, students took a 100-question multiple-choice quiz covering the key knowledge points from those chapters. No significant difference was observed in quiz scores between the two groups (Table 1). The control group (*n* = 30) continued with traditional didactic lectures, while the other group (*n* = 30), designated as the innovation group, participated in an innovative program integrating CBL and CM in the study of material metabolism chapters. The innovation group was further divided into five subgroups (*n* = 6 per subgroup). Both groups were taught by the same instructor throughout the study period, ensuring equivalent teaching time and consistent instructional quality. This study was conducted from September 2023 to January 2024 and strictly adhered to the ethical principles outlined in the World Medical Association Declaration of Helsinki. It was approved by the Human Ethics Committee of Hunan University of Medicine, China.

**Table 1:** The comparison of examination scores

group	*n*	Quiz scores	*t*	95%CI	Effect size (Cohen's *d*)	*P* value	Final examination scores	*t*	95%CI	Effect size (Cohen's *d*)	*P* value
Control group	30	75.60 ± 7.74	−0.487	−5.28—3.22	0.125	0.628	68.00 ± 13.83	−3.349	−16.14—−4.06	0.870	0.001
Inovative group	30	75.63 ± 8.68					78.10 ± 9.03				

Differences in examination scores between two groups were analyzed by independent-samples *t*-test and the results were expressed as mean ± SD. *P* < 0.05 was considered statistically significant

### Study design

The innovative program consisted of two main components. The first component was a student training session, which included: 1) an introduction to the theoretical foundations of CM, 2) step-by-step instruction on constructing concept maps, and 3) guidance on applying CM in medical education contexts. The second component comprised four modules focused on concept mapping through clinical case analysis. The cases were constructed in collaboration with clinicians, based on common medical challenges that students are likely to encounter after graduation, with a primary emphasis on material metabolism and related diseases caused by metabolic disorders. The cases were concise, meticulously structured, and accurately framed, featuring clearly articulated learning objectives and a systematic, contextually appropriate discussion of questions and key concepts designed to align with these objectives (see Table 2).

**Table 2:** An example of a clinical case analysis module

Topic: Diabetic Ketoacidosis‌ (DKA)
A 50-year-old female with a 12-year history of diabetes was admitted to the hospital in coma with breath smelling of rotten apples. Physical examination showed blood pressure of 12/5.3 kPa (90/40 mmHg), pulse rate of 110 beats per minute, respiratory rate of 28 breaths per minute, and urine glucose and ketones both + + +. She is suspected of having DKA Questions: 1) What is the biochemical mechanism behind the "fruity" or "rotten apple" odor in the patient’s breath, and which ketone body is primarily responsible for this clinical sign? 2) Why does insulin deficiency lead to increased lipolysis and fatty acid oxidation, and how does this shift in metabolism result in excessive ketone body production in the liver? 3) Under what biochemical conditions does acetyl-CoA divert from the TCA cycle to ketogenesis, and how do hormonal imbalances (e.g., low insulin, high glucagon) regulate this metabolic switch? 4) How do the accumulation of β-hydroxybutyrate and acetoacetate lead to high-anion-gap metabolic acidosis, and what are the physiological consequences of this acid–base disturbance? 5) What is the role of osmotic diuresis in DKA, and how does hyperglycemia exceeding the renal threshold affect electrolyte and fluid balance at the molecular level? 6) How does the body attempt to compensate for metabolic acidosis in DKA, and what is the biochemical basis of Kussmaul respiration? 7) What metabolic changes occur during prolonged fasting or uncontrolled diabetes that make the liver a net producer of ketone bodies rather than glucose? Key concepts: Diabetes; Diabetic Ketoacidosis; Ketone Body; β-Oxidation; Electrolyte Imbalance; Ketonemia; Kussmaul Respiration; Ketoacidosis; Dehydration; Fruity Breath; Osmotic Diuresis Specific Learning Objectives: 1) Explain the critical role of insulin in promoting glucose uptake and inhibiting gluconeogenesis and lipolysis, as well as the cascade of metabolic disturbances caused by its absolute or relative deficiency in DKA Illustrate the biochemical mechanisms by which fatty acid β-oxidation in the liver generates acetyl-CoA, leading to ketogenesis (acetoacetate, β-hydroxybutyrate, acetone), and the pathways of ketone body utilization in peripheral tissues Fully understand the sources and fates of acetyl-CoA 4) Analyze how the accumulation of organic acids such as acetoacetate and β-hydroxybutyrate triggers respiratory compensation (Kussmaul respiration) 5) Understand the biochemical link between hyperglycemia and osmotic diuresis 6) Be familiar with the chemical reaction mechanisms and clinical interpretation of urine glucose detection using Benedict's test or glucose oxidase method, and ketone body detection 7) Clarify the biochemical mechanisms of electrolyte imbalance due to osmotic diuresis and acidosis 8) Clarify the biochemical mechanisms of coma due to ketoacidosis

**Fig. 1:**
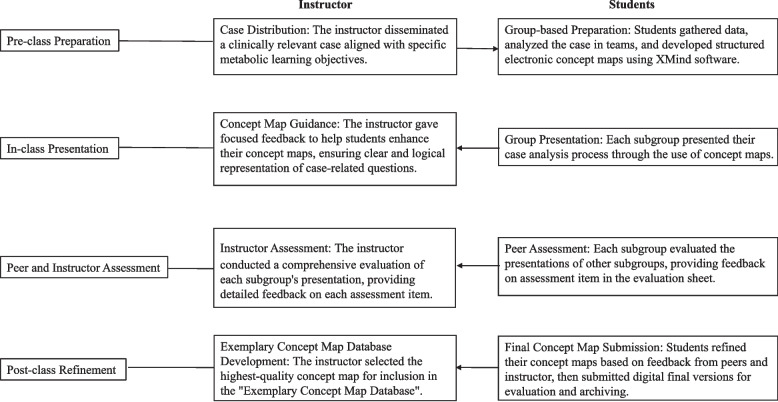
A procedure of the clinical case analysis session

The clinical case analysis module was conducted through the following structured procedure (Fig. 1):

1. Pre-class Preparation

Students received a clinical case the week before the module to review and become familiar with new concepts related to substance metabolism and clinical practice. We provided several key concepts to guide map construction without imposing constraints on the overall structure, and allowed student groups the freedom to organize their concept maps around these given concepts, as reported by Slieman [1]. Each subgroup independently researched the case and collaboratively developed electronic concept maps using XMind software (https://xmind.cn/).

2) In-class presentation (70 min)

A representative from each subgroup systematically presented and analyzed the clinical case using their concept maps during class sessions.

3) Peer and instructor assessment (20 min)

Subgroup presentations were evaluated by other subgroups and the instructor using a standardized evaluation sheet (Table 3).

**Table 3:** Evaluation sheet for case analysis

Assessment item	Assessment content	Scores				
		Strongly agree	Agree	Neither agree nor disagree	disagree	Strongly Disagree
Preparatory Work for the Presentation	All necessary materials and resources were prepared in advance	5	4	3	2	1
Case Analysis Presentation	Fluency & Expressiveness: The presenter delivered the clinical case analysis with remarkable fluency, maintaining a natural and elegant presentation style	5	4	3	2	1
	Clarity of Thought: The case was analyzed with exceptional clarity, demonstrating a logical and structured approach to problem-solving	5	4	3	2	1
	‌Concept Integration‌: The case was seamlessly integrated with the concept map, ensuring a coherent and interrelated framework	5	4	3	2	1
	‌Concept Map Design‌: The concept map effectively visualized the case, presenting hierarchical relationships between key concepts	5	4	3	2	1
	‌Conclusion Validity‌: The analysis, grounded in the concept map, yielded a well-supported and clinically sound conclusion	5	4	3	2	1
Presentation Effectiveness & Impact	‌Identity Construction‌: The presentation fostered a strong sense of identity among the audience, enabling them to connect with the case on a personal and professional level	5	4	3	2	1
	‌Engagement & Understanding‌: The presentation is highly engaging, captivating the audience's attention while facilitating a deeper understanding of the case through clear communication and visual aids	5	4	3	2	1

4) Post-class refinement

Following feedback, each subgroup further refined their concept maps and submitted the revised versions the day after the class session. The highest-quality map was selected for inclusion in a concept-map resource database, an open-access learning tool designed to support students' independent review and study.

### Data evaluation and statistical analysis

The combined effects of CBL and CM on student learning outcomes were evaluated through two complementary approaches:

1. Final examination performance

Both cohorts completed an identical 200-item multiple-choice final examination, with scores systematically compared to assess knowledge acquisition and retention. The final examination was administered one month after students completed the CBL-CM hybrid approach, covering the key knowledge points from the first five chapters and the material metabolism sections. Questions assessing understanding and mastery of fundamental concepts accounted for 60%, while those requiring comprehensive analysis and case-based analysis accounted for 40%. All questions were randomly selected from an early-established biochemistry exam question bank. These items were meticulously aligned with students’ learning objectives and the course syllabus, and the selection was conducted by a teacher who was not involved in this study, ensuring an unbiased and objective assessment.

2) Innovative group perceptions

All 30 members of the innovation group completed a feedback questionnaire consisting of an 8-item, 5-point Likert scale and two open-ended questions. The scale was designed to assess students' perceptions of the combined CBL and CM approach as shown in Table 4. It was developed based on guidelines established by Surapaneni et al. [7] and Chiou et al. [8]. While the open-ended questions addressed their willingness to apply these methods in future studies/professional practice and suggestions for program improvement.

**Table 4:** Students' feedback on the integrated effects of the CBL and CM approach (*n* = 30)

Item	Scores
The integration of CBL with CM effectively fosters self-directed learning	4.83 ± 0.38
I enjoy the integration of CBL and CM	4.93 ± 0.25
The integration of CBL with CM significantly improves knowledge comprehension and long-term retention	4.90 ± 0.31
The integration of CBL with CM effectively facilitates knowledge integration	4.87 ± 0.35
The integration of CBL with CM significantly enhances analytical skills	4.20 ± 0.76
The integration of CBL with CM significantly enhances logical thinking ability	4.97 ± 0.18
The integration of CBL with CM significantly enhances knowledge application ability	4.37 ± 0.67
The integration of CBL with CM effectively bridges the gap between foundational knowledge and clinical application	4.83 ± 0.38

Scores were expressed as mean ± SD

Data were represented as mean ± SD. Differences in examination scores between two groups were analyzed by independent-samples *t*-test. A value of *P* < 0.05 was considered statistically significant. All data analysis were performed with SPSS version 22.0 (SPSS, Inc., Chicago, Illinois, United States).

## Results

**Fig. 2:**
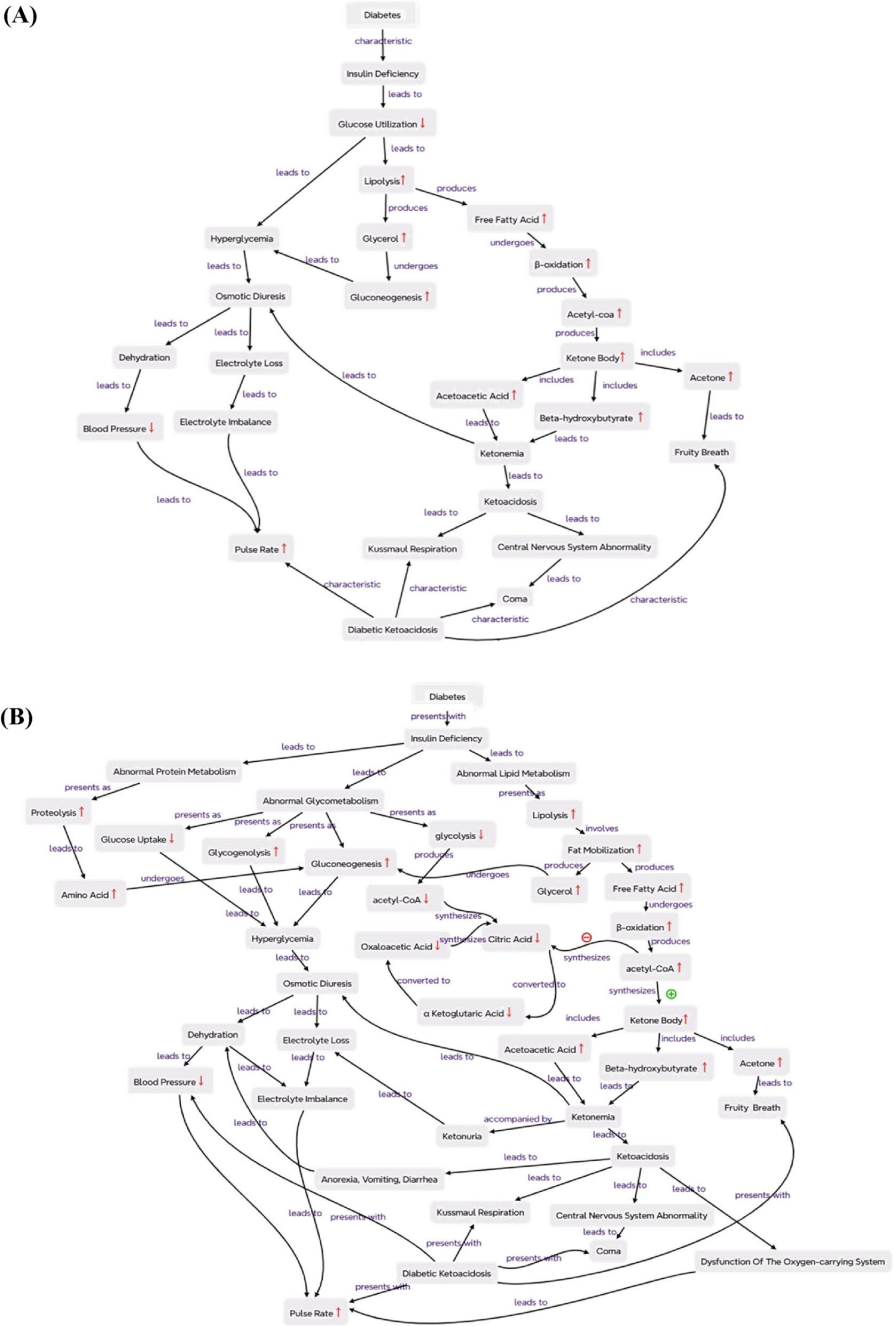
Representative concept maps created by student group using XMind software. **A** Student group concept map from the initial stage showing simplistic, or missing links. **B** Student group concept map at the final stage showing more accurate, detailed, and comprehensive connections

To assess the combined impact of CBL and CM on student achievement, we compared final examination scores between the control and innovative groups. The results demonstrated that both groups performed similarly on the pre-intervention quiz, confirming baseline equivalence. However, students who participated in the innovative program achieved significantly higher final examination scores than those who received traditional lectures (78.10 ± 9.03 vs. 68.00 ± 13.83) (Table 1). Improvement in the construction of concept maps can also be observed in representative maps created by students the innovation group (Fig. 2).

The analysis of students' perceptions regarding the combined use of CBL and CM revealed overwhelmingly positive results. The vast majority of students approved of the effectiveness of this combined approach in the biochemistry curriculum (Table 4), with their preference for the CBL-CM combination reaching a high average score of 4.93. Notably, the highest-rated item (mean = 4.97) concerned the extent to which the integrated use of CBL and CM enhanced their logical thinking skills. The item assessing the approach's effectiveness in bridging the gap between basic science and clinical practice also received a high score of 4.83. Furthermore, an impressive 90% (27/30) of students indicated they would apply both CBL and CM in their future studies and professional practice.

Moreover, the students put forward two key suggestions for improving the innovative program. First, they felt the process of creating concept maps using software to be time-consuming, and sometimes the electronic outputs failed to fully meet their expectations. They expressed a desire to have concept maps presented in more diverse formats rather than just digital versions. Second, while each subgroup was eager to present its case analysis using concept maps, the sequential presentation format (with five groups waiting in line) created challenges. Some groups found that when their clinical case analyses were similar to those presented earlier, their enthusiasm for presentation diminished, as they felt their unique perspectives might be overshadowed or repeated. This sequential presentation method also risked influencing the analyses of later-presenting groups. To address this, the students suggested implementing a more effective approach to enhance the quality and fairness of case presentations.

## Discussion

The present study demonstrated that, compared to traditional didactic instruction, the innovative program integrating CM and CBL significantly enhanced student learning outcomes. This finding aligns with the observations of Wang et al., who reported that the combined application of CM and CBL in biochemistry courses effectively stimulated students' enthusiasm for learning and promoted self-learning abilities, logical reasoning, and scientific information processing skills [9]. Meanwhile, in this study, the majority of students participating in our innovative program clearly expressed their preference for the CBL-CM combination. When compared to our previous evaluation of CM implementation alone [5], students showed a distinct preference for the integrated CBL-CM approach, particularly highlighting its superior effectiveness in enhancing logical thinking and bridging basic theory with clinical practice, as reflected in their significantly higher evaluation scores.

Students' distinct preference for the CBL-CM method was not accidental. It stemmed from the method's multi-faceted optimization of the learning process. Firstly, the CBL-CM method stimulated learning interest. Traditional teaching approaches are often teacher-centered, with students passively receiving knowledge and easily becoming bored. In contrast, the CBL-CM method introduces real-life cases, integrating abstract theoretical knowledge with vivid clinical practice, making the learning process both engaging and intellectually challenging. In our study, students showed greater enthusiasm when analyzing complex cases, as they were no longer merely memorizing isolated facts but actively exploring knowledge through solving authentic clinical problems. Secondly, the CBL-CM method provides students with ample opportunities for independent exploration. Learners must independently gather information, analyze problems, and develop solutions, thereby gradually enhancing their information-acquisition, critical-thinking, and decision-making skills. Moreover, when students construct concept maps based on clinical cases, they break down complex content into smaller parts. This allows them to conduct in-depth analysis and organization of knowledge, integrate newly-acquired learning with their existing cognitive structures, and engage in critical thinking by questioning assumptions, evaluating evidence, and drawing well-reasoned conclusions. Collectively, these processes facilitate the discovery of internal knowledge connections, leading to enhanced understanding of the content, improved logical reasoning, and better inductive summarization. This might partially account for why the CBL- CM method has shown significant advantages in enhancing logical thinking. Finally, traditional teaching struggles to link theory and practice, hindering students' ability to apply classroom knowledge in clinical settings. The CBL-CM method, however, integrates real-life cases, enabling students to learn and apply knowledge while solving authentic problems, thereby bridging theory and practice. This approach deepens students' understanding and builds practical experience, better preparing them for professional demands.

Therefore, the integration of CBL and CM holds significant potential for fostering medical students' clinical competencies. However, it also presents a formidable challenge that how to effectively combine these methods and maximize their synergistic benefits. Based on our implementation experience and students' feedback, we offer the following recommendations: 1) Case selection is crucial. Cases should be carefully designed to target key learning challenges with an appropriate level of complexity. Cases that generate divergent viewpoints among students are particularly effective in driving learning motivation, stimulating a sense of challenge, and enhancing participation. 2) Sufficient time must be allocated for students to thoroughly analyze cases and systematically construct concept maps, ensuring they arrive in class fully prepared to engage in meaningful discussions. 3) It is recommended to encourage students to transcend the constraints of traditional PowerPoint by adopting diverse presentation formats, including wall-mounted concept maps and blackboard illustrations. Such variety in visual expression facilitates a more seamless integration of CM with CBL, while accommodating diverse learning preferences and fostering richer classroom interaction. Through this approach, concept maps evolve beyond static knowledge organizers into dynamic mediums that actively cultivate clinical reasoning abilities. 4) Instructors can facilitate real-time group scoring and interactive voting by leveraging smart teaching platforms such as Rain Classroom, thereby enhancing the objectivity and fairness of formative assessment. 5) Where feasible, the utilization of smart classrooms is recommended for implementing the combined methodology of CBL and CM. The round-table configuration promotes more effective group discussions and collaborative interactions, while the multi-screen setup facilitates efficient comparison and dynamic presentation across different group case analyses.

## Conclusion

Our findings demonstrated that the integration of CBL and CM significantly enhanced biochemistry learning outcomes. Student feedback on this combined approach was overwhelmingly positive. They particularly valued the combined use of CBL and CM for enhancing their logical thinking skills and bridging the gap between basic science and clinical practice. However, this study also has several limitations. First, the sample size was small, and limited number of clinical case analysis modules (only four) resulted in a short intervention duration. Furthermore, Implementing an effective CBL-CM hybrid instructional model requires substantial preparation time from both instructors and students. More importantly, it is crucial to design and implement the integration strategy according to specific curriculum characteristics, student profiles, and institutional resources to maximize its educational potential.

## Data Availability

Datasets supporting the conclusions of this article are included within the article.

## Abbreviations

CM: Concept mapping
CBL: Case-based learning
